# Real-World Evidence on the Use of Traditional Korean Medicine in Managing Intervertebral Disc Disease

**DOI:** 10.3390/healthcare13212661

**Published:** 2025-10-22

**Authors:** Boram Lee, Jun-Su Jang, Mi Hong Yim

**Affiliations:** 1 KM Science Research Division, Korea Institute of Oriental Medicine, Daejeon 34054, Republic of Korea; qhfka9357@kiom.re.kr; 2 Digital Health Research Division, Korea Institute of Oriental Medicine, Daejeon 34054, Republic of Korea; junsu.jang@kiom.re.kr

**Keywords:** intervertebral disc disease, traditional medicine, Korean medicine, healthcare use, Korea Health Panel Survey, Andersen’s behavioral model

## Abstract

**Background/Objectives:** Korean medicine healthcare (KMHC), a form of traditional medicine including acupuncture and herbal medicine, is widely utilized by patients with intervertebral disc disease (IVDD). With the increasing use of real-world evidence (RWE) in the medical field, this study aims to derive RWE on KMHC utilization and its associated factors in patients with IVDD. **Methods:** Data from 495 individuals who received outpatient healthcare for IVDD regardless of the purpose such as treatment, examination, rehabilitation, monitoring, or prescription were analyzed using the 2022 Korea Health Panel Survey (KHPS). Multinomial logistic regression analyses were performed to identify factors associated with healthcare use for IVDD. Regression models were constructed by sequentially adding predisposing, enabling, and need factors following Andersen’s behavioral model. All statistical analyses accounted for the complex survey design of the KHPS using survey sampling weights. **Results:** Individuals aged 45–59 years were less likely to use both KMHC and conventional medicine healthcare (CMHC) for IVDD compared to those aged 19–44 years (adjusted odds ratio [95% confidence interval], 0.28 [0.09, 0.89]). People with disabilities showed lower utilization of both KMHC and CMHC for IVDD compared to those without disabilities (0.27 [0.09, 0.81]). Individuals who were employed (2.37 [1.06, 5.3]) or perceived their health status as fair (3.05 [1.17, 8]) or poor/very poor (6.13 [2.04, 18.45]) were more inclined to use both KMHC and CMHC for IVDD. Individuals who engaged in regular physical activities (2.65 [1.19, 5.9]) or had shoulder joint diseases (3.71 [1.22, 11.29]) or other spine-related diseases (2.63 [1.16, 5.96]) were more inclined to use KMHC-only for IVDD. **Conclusions:** This study identified significant demographic and health-related factors influencing KMHC utilization for IVDD. These findings emphasize the need for tailored healthcare policies regarding KMHC for IVDD for effective resource distribution.

## 1. Introduction

Intervertebral disc disease (IVDD) is a leading cause of disability globally, with its prevalence increasing with an aging population [1]. In developed countries, it affects approximately 5% of the population annually, leading to a significant socioeconomic burden worldwide [2,3]. The most common forms of IVDD are degenerative disc disease (DDD) and prolapsed intervertebral disc, with primary symptoms including back, neck, and radicular pain [1]. However, some DDDs are asymptomatic, and magnetic resonance imaging (MRI) has shown disc abnormalities in up to 80% of asymptomatic individuals, suggesting that the true prevalence of IVDD is estimated to be significantly higher [4]. Conventional medicine healthcare (CMHC) strategies for managing IVDD vary depending on the disease type and severity. They can be broadly divided into conservative treatments—such as medication, physiotherapy, and epidural injections—and surgical treatments [1,4]. In particular, the number of outpatients receiving CMHC for dorsalgia, a major symptom of IVDD in South Korea, was 5,732,221 in 2023, ranking 8th in frequency among all conditions [5]. However, many patients do not respond to conservative treatment, while surgical treatment has limitations such as higher recurrence and revision rates, lack of long-term effectiveness, and complication risk [4,6,7,8].

Korean medicine healthcare (KMHC), such as herbal medicine and acupuncture, corresponding to traditional East Asian medicine treatment, has been actively employed in treating IVDD. In the clinical medical field in Korea, studies show that dorsalgia—a major symptom of IVDD—is the most common symptom receiving KMHC [5]. Additionally, a pilot project is set to provide health insurance coverage for herbal decoction for lumbar disc herniation, one of the IVDDs, from April 2024 to December 2026 [5,9]. Several clinical studies show the effectiveness of KMHC in treating IVDD [10,11,12]. Furthermore, KMHC has demonstrated benefits in patients who failed back surgery, with significant improvements in pain management and physical function, and satisfaction was also high [13].

Real-world evidence (RWE) refers to clinical evidence regarding the usage, benefits, or risks of a medical product derived from analysis of real-world data, such as electronic health records, insurance claims, or national health surveys [14]. The use of RWE is rapidly increasing in the medical field in recent times, and it is being used in various approaches such as drug development, medical policy decision-making, and treatment outcome analysis [15]. Analyzing RWE related to healthcare utilization offers accurate insights into treatment effectiveness based on routine clinical practice by including diverse patient populations that are often excluded from clinical trials. This approach enhances the generalizability of findings. Additionally, patient preferences and trends can be identified using big data-based healthcare utilization status. This insight can inform evidence-based decision-making by policymakers on resource allocation and healthcare utilization guidelines.

However, to our knowledge, although KMHC is actively used in patients with IVDD owing to limitations in CMHC and patient preferences [5,9], real-world data on its usage status and factors have not been analyzed. Although a previous study [9] examined KMHC utilization using the 2017 Korean Medicine Utilization and Herbal Medicine Consumption Survey data targeting 5000 Korean citizens, the analysis was limited to reporting simple utilization rates according to the presence or absence of several musculoskeletal diseases. That study did not examine factors associated with KMHC use and did not specifically focus on the population with IVDD, limiting insights into this patient group. Therefore, this study aims to derive RWE on the current status and factors associated with KMHC use in patients with IVDD.

## 2. Materials and Methods

### 2.1. Data Source and Study Participants

In this study, data from the 2022 Korea Health Panel Survey (KHPS) conducted by the Korea Institute for Health and Social Affairs and the National Health Insurance Service were used. The KHPS is an annual nationwide panel survey that collects data on healthcare utilization, healthcare costs, demographic characteristics, social and economic characteristics, health status, and health behavior. Data collection was conducted using the Computer Assisted Personal Interviewing method, involving a face-to-face interview where the researcher visited target households and conducted the survey using a laptop while administering the questionnaire. To ensure national representativeness, the KHPS sample was selected using a stratified two-stage cluster sampling method. KHPS data are anonymized and publicly available on the official website (https://www.khp.re.kr, accessed on 10 June 2024). This study received a protocol review exemption, which was approved by the Institutional Review Board of the Korea Institute of Oriental Medicine (IRB No. I-2403/003-004).

**Figure 1:**
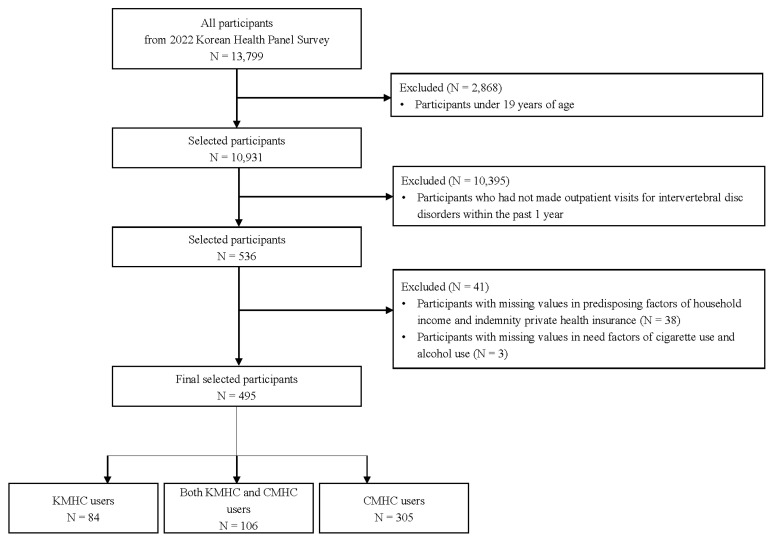
Flow chart of study sample selection. CMHC, conventional medicine healthcare; KMHC, Korean medicine healthcare.

Data from the 2022 Korea Health Panel Survey (KHPS) were used for this study. Participants were included if they were adults (≥19 years) who received outpatient care for IVDD within the past year for any purpose such as treatment, examination, rehabilitation, monitoring, or prescription. Among the 13,799 respondents in the 2022 KHPS, 10,931 adults aged 19 years or older were initially selected. Of these, 536 participants who had made outpatient visits for IVDD within the past year were initially identified. After excluding 41 participants with missing values in the survey data, the final sample included 495 participants, comprising 84 KMHC-only users, 106 both KMHC and CMHC users, and 305 CMHC-only users (Figure 1).

### 2.2. Outcome and Explanatory Variables

The primary outcome was whether individuals had utilized KMHC for IVDD in an outpatient visit within the past 1 year. This was classified into three categories, namely KMHC-only, both KMHC and CMHC, and CMHC-only uses. The participants were asked to report their healthcare utilization history over the past year and provide receipts for these visits. The researchers then reviewed the healthcare usage records and receipts.

The explanatory variables for KMHC use for outpatient visits for IVDD were determined using Andersen’s behavioral model of health service use [16,17,18,19]. These variables were classified into three categories, namely predisposing, enabling, and need factors, following their theoretical framework. Predisposing, enabling, and need factors refer to individual characteristics before using health services, the ability of the individual to access health services, and the perceived and assessed health status, respectively [20,21,22,23]. Predisposing factors included sex, age, region, education level, and marital status. Enabling factors included the number of household members, household income, private indemnity health insurance, and employment status. Need factors included disability (as registered with the Korean Ministry of Health and Welfare), perceived health status, perceived stress, depressed mood, regular physical activities, alcohol consumption, cigarette use, body mass index, presence of arthritis, shoulder joint diseases, and other spine-related diseases, and the number of chronic diseases. The number of chronic diseases was categorized according to the number of the following diseases: hypertension, diabetes mellitus, liver disease, malignant neoplasm, cardio-cerebrovascular disease, chronic lower respiratory disease, thyroid dysfunction, dementia, and chronic renal failure.

### 2.3. Statistical Analysis

All statistical analyses utilized survey sampling weights to account for the complex survey design of the KHPS, ensuring that estimates are representative of the target population, the Korean populace. All statistical analyses were conducted using R version 4.4.1 (R Foundation for Statistical Computing, Vienna, Austria) and the complex samples procedure in IBM SPSS Statistics for Windows, version 29.0 (IBM Corp., Armonk, NY, USA). For data management, transformation, and visualization, we additionally utilized several R packages such as base R, haven, stringr, dplyr, tidyr, magrittr, purrr, ggplot2, and scales. All statistical analyses were two-tailed, and statistical significance was defined as *p* < 0.05. The same significance threshold was applied consistently across all analyses and tables.

To compare differences in general characteristics among healthcare user groups for IVDD, Rao-Scott Chi-square tests were employed for complex survey design. Results were summarized as unweighted frequencies and weighted column proportions. To identify factors associated with healthcare utilization for IVDD, multinomial logistic regression analyses were conducted using sampling weights as the outcome was classified into three categories: KMHC-only use, both KMHC and CMHC use, and CMHC-only use [24]. First, crude analyses were conducted using multinomial logistic regression with an individual explanatory variable. Three models were then constructed using multinomial logistic regression with combined explanatory variables by sequentially adding predisposing, enabling, and need factors. The results were presented as odds ratios with 95% confidence intervals. To assess differences in healthcare services among healthcare user groups for IVDD, Rao-Scott Chi-square tests and general linear model analyses were used for categorical and continuous variables, respectively. The results were summarized as unweighted frequencies with weighted column proportions for categorical variables and as means with standard errors for continuous variables.

## 3. Results

### 3.1. General Characteristics of Users of Healthcare for Intervertebral Disc Disease

In total, 495 individuals participated in this study, including 188 men and 307 women. Significant differences were observed among the three healthcare user groups—KMHC-only, both KMHC and CMHC, and CMHC-only users—in age, region, regular physical activities, shoulder joint diseases, and other spine-related diseases. The proportion of individuals aged 45–59 years was the highest among CMHC-only users (weighted proportion: 47.1%), whereas the proportion of individuals aged 60–74 years was the highest among both KMHC and CMHC users (33.66%) and KMHC-only users (28.95%). The proportion of residents in Daejeon/Sejong/Chungcheong was the lowest among CMHC-only users (8.79%) and KMHC-only users (14.18%), while the proportion of residents in Gwangju/Jeolla/Jeju was the lowest in both KMHC and CMHC users (15.6%). The proportion of individuals engaging in regular physical activities was higher in KMHC-only users (63.49%) and both KMHC and CMHC users (61%) than in CMHC-only users (46.84%). The proportion of individuals with shoulder joint diseases was lower in both KMHC and CMHC users (3.75%) and CMHC-only users (4.13%) than in KMHC-only users (15.1%). The proportion of individuals with other spine-related diseases was lower in both KMHC and CMHC users (10.3%) and CMHC-only users (14.24%) than in KMHC-only users (30.2%) (Table 1).

### 3.2. Factors Associated with Healthcare Use for Intervertebral Disc Disease

In crude analyses, significant associations were observed between individual explanatory variables and healthcare use for IVDD. Both KMHC and CMHC use relative to CMHC-only use was observed to be significantly associated with age (45–59, crude odds ratio [95% confidence interval], 0.4 [0.16, 0.96]), disability (0.32 [0.11, 0.9]), and perceived health status (Poor/Very poor, 2.93 [1.16, 7.41]). KMHC-only use relative to CMHC-only use was identified to be significantly associated with region (Gwangju/Jeolla/Jeju, 3.48 [1.57, 7.7]), marital status (Widowed/Divorced/Separated/Never married, 2.25 [1.1, 4.59]), number of household members (4 or more, 0.32 [0.13, 0.82]), regular physical activities (1.97 [1.01, 3.84]), shoulder joint diseases (4.13 [1.56, 10.94]), and other spine-related diseases (2.61 [1.25, 5.44]). There were some changes in the variables associated with healthcare use for IVDD after adjusting sequentially for predisposing, enabling, and need factors. In adjusted model 1, adjusted for combined explanatory variables of predisposing factors, age (45–59, adjusted odds ratio [95% confidence interval], 0.33 [0.12, 0.93]), and education level (Middle/High school, 2.21 [1.03, 4.73]) were significantly associated with both KMHC and CMHC use relative to that of CMHC-only use, while the region (Gwangju/Jeolla/Jeju, 3.72 [1.57, 8.77]) was associated with KMHC-only use relative to that of CMHC-only use. In adjusted model 2, adjusted for combined explanatory variables of predisposing and enabling factors, both KMHC and CMHC use relative to CMHC-only use was observed to be significantly associated with age (45–59, 0.33 [0.12, 0.91]), while KMHC-only use relative to CMHC-only use was observed to be significantly associated with age (75 or older, 4.74 [1.11, 20.19]) and region (Gwangju/Jeolla/Jeju, 5.37 [2.13, 13.5]) (Table S1).

**Figure 2:**
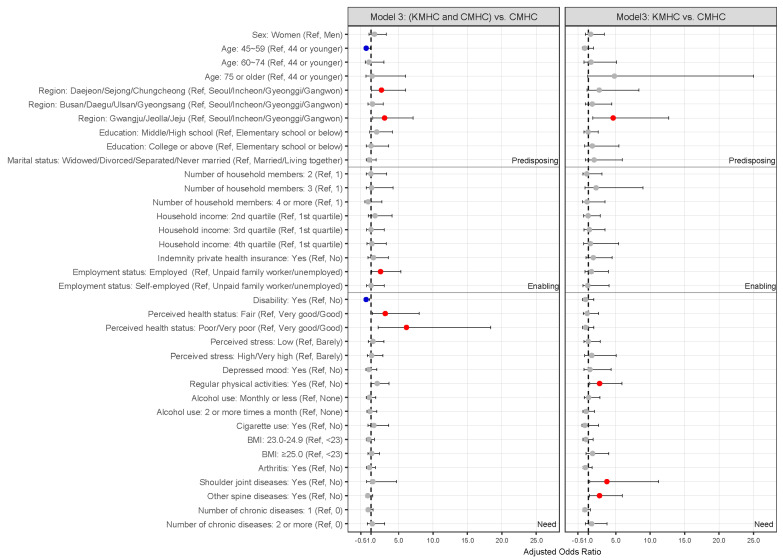
Odds ratios plot with 95% confidence intervals for factors associated with healthcare use for IVDD in fully adjusted model. Red dots indicate statistically significant positive associations (odds ratio > 1), blue dots indicate statistically significant negative associations (odds ratio < 1), and gray dots indicate non-significant associations. BMI, body mass index; CMHC, conventional medicine healthcare; KMHC, Korean medicine healthcare; IVDD, intervertebral disc disease.

In a fully adjusted model for combined explanatory variables of predisposing, enabling, and need factors, both KMHC and CMHC uses relative to CMHC-only uses were significantly associated with age, region, employment status, disability, and perceived health status. Individuals aged 45–59 years were less inclined to use both KMHC and CMHC for IVDD compared to those aged 19–44 years (0.28 [0.09, 0.89]). Residents in Daejeon/Sejong/Chungcheong (2.5 [1.04, 6.02]) and Gwangju/Jeolla/Jeju (2.95 [1.23, 7.09]) were more inclined to use both KMHC and CMHC in IVDD compared to residents in Seoul/Incheon/Gyeonggi/Gangwon. The employed had a higher tendency to use both KMHC and CMHC for IVDD compared to unpaid family workers or the unemployed (2.37 [1.06, 5.3]). People with disabilities were less inclined to use both KMHC and CMHC for IVDD compared to those without disabilities (0.27 [0.09, 0.81]). Individuals who perceived their health status as fair (3.05 [1.17, 8]) or poor/very poor (6.13 [2.04, 18.45]) were more inclined to use both KMHC and CMHC for IVDD compared to those who perceived their health status as very good/good. KMHC-only use relative to CMHC-only use was significantly associated with the region, regular physical activities, shoulder joint diseases, and other spine-related diseases. Residents in Gwangju/Jeolla/Jeju exhibited a higher tendency to use KMHC-only for IVDD compared to those who live in Seoul/Incheon/Gyeonggi/Gangwon (4.61 [1.67, 12.75]). Individuals who engaged in regular physical activities were more inclined to use KMHC-only for IVDD compared to those who did not engage in regular physical activities (2.65 [1.19, 5.9]). Individuals who were suffering from musculoskeletal disorders, including shoulder joint diseases (3.71 [1.22, 11.29]) and other spine-related diseases (2.63 [1.16, 5.96]) exhibited a higher tendency to use KMHC-only for IVDD compared to individuals without these conditions (Table 2 and Figure 2).

### 3.3. Healthcare Services for Patients with Intervertebral Disc Disease

Among KMHC services for IVDD, the most commonly performed treatment was acupuncture (92.81%), followed by physical therapy (90.54%), cupping therapy (27.46%), pharmacopuncture (27.35%), herbal decoction (13.94%), and chuna manual therapy (13.74%). In KMHC-only users, acupuncture (91.93%) was the most prevalent treatment, followed by physical (84.79%) and cupping therapies (30%). In both KMHC and CMHC users, physical therapy (93.85%) was the most commonly used, followed by acupuncture (93.32%) and pharmacopuncture (31.61%). No statistically significant differences were observed between both KMHC and CMHC users and KMHC-only users in Korean medicine healthcare services, copayment per visit, or annual number of healthcare uses for IVDD (Table 3).

Among CMHC services for IVDD, the most commonly used healthcare service was physical therapy (66.41%), followed by high-cost imaging tests (26.48%), manual therapy (14.42%), and intravenous injections (9.98%). In both KMHC and CMHC users, physical therapy (78.51%) was the most frequently used, followed by high-cost imaging tests (40.27%) and intravenous injections (17.23%). In CMHC-only users, physical therapy (62.41%) was the most prevalent treatment, followed by high-cost imaging tests (21.92%) and manual therapy (16.38%). Significant differences in CMHC services (high-cost imaging tests, intravenous injections, and physical therapy) were observed between both KMHC and CMHC users and CMHC-only users. Conversely, no significant differences were observed in copayment per visit or the annual number of healthcare uses for IVDD (Table 4).

## 4. Discussion

This study aimed to derive RWE on the utilization of KMHC among patients with IIVDD and to identify the factors influencing KMHC use. Using nationally representative data from the 2022 KHPS, 495 patients who received outpatient medical care for IVDD within the past 1 year were analyzed. We found that employment status, perceived health, physical activity, and comorbid musculoskeletal conditions were key determinants of KMHC utilization. While a previous study shows the factors affecting KMHC use in patients with musculoskeletal disorders, the study was conducted in 2009 and analyzed overall musculoskeletal disorders rather than specific diseases [25]. Consequently, its current applicability has limitations.

According to Andersen’s theoretical framework, the sequential input of predisposing, enabling, and need factors in healthcare utilization models reflects their temporal order [20]. An individual’s use of medical services is based on basic predisposition factors, with additional enabling factors increasing the likelihood of use, and ultimately, need factors that lead to healthcare utilization [20]. By constructing models sequentially, the independent and step-by-step influence of each factor can be identified [20,26]. Additionally, the complex mechanisms of medical service utilization can be better understood by observing changes in significant variables across models [20,26]. In our study, education level was significantly associated with both KMHC and CMHC use relative to CMHC-only use in adjusted model 1. However, it was not significant in adjusted model 2 with additional enabling factors, suggesting that the effect of education level operates through enabling factors such as income or insurance. Variables that remain significant in the final model can be prioritized for policy intervention.

In fully adjusted model, among the predisposing factors influencing KMHC use in patients with IVDD, individuals aged 45–59 years were less inclined to use both KMHC and CMHC relative to CMHC-only compared to those aged 19–44 years. This may reflect a greater health consciousness among younger adults than among middle-aged adults and thus attempting more active and diverse treatments such as using both KMHC and CMHC [27,28]. These findings suggest that targeted awareness campaigns for middle-aged adults may help promote more informed and diversified healthcare choices among this group. Additionally, while no statistically significant difference was observed, patients with IVDD aged ≥75 years were more inclined to use KMHC-only compared to those aged 19–44 years. This observation may be explained by the presence of musculoskeletal conditions (e.g., osteoarthritis, disc-related disease, and back pain) as common reasons for KMHC use among older patients [29]. However, our study is meaningful in that it showed that age-related trends can be observed even when controlling for other chronic diseases in IVDD patients. Moreover, individuals > 75 years old may prefer KMHC—a non-surgical and relatively safe treatment option—owing to concerns about surgical treatment risks and medication-related adverse events [30,31]. Additionally, patients with IVDD residing in Daejeon/Sejong/Chungcheong were more inclined to use both KMHC and CMHC compared to those in Seoul/Incheon/Gyeonggi/Gangwon, and patients residing in Gwangju/Jeolla/Jeju tended to use KMHC-only or both KMHC and CMHC compared to those in Seoul/Incheon/Gyeonggi/Gangwon. The regional pattern observed in this study contrasts with earlier nationwide findings that reported greater KHMC use in Seoul [32]. This discrepancy may be explained by improved accessibility to Korean medicine services in regional areas, higher cultural preference for traditional medicine among older populations, and recent policy initiatives by local governments expanding support for KMHC. However, in that study [32], the results were analyzed regardless of disease, whereas, in this study, patients with IVDD were targeted, suggesting that regional preferences for KMHC may vary by region based on the disease of the patient.

As an enabling factor, employed patients with IVDD tended to use both KMHC and CMHC relative to CMHC-only more, compared to unpaid family workers or unemployed patients. This may be attributed to the increased medical costs as treatment types increased in both KMHC and CMHC users [33]. In this study, our findings indicated that the annual copayment of medical expenses for KMHC and CMHC utilization for IVDD was higher among both KMHC and CMHC users than among KMHC-only and CMHC-only users.

As a need factor, patients with IVDD with disabilities registered under the Korean Ministry of Health and Welfare—such as physical disability, developmental disability, and epilepsy—were less inclined to use both KMHC and CMHC relative to CMHC-only compared to those without disabilities. For patients with severe disabilities, engaging in normal economic activities might be challenging, while the cost of treating the disabilities might be significantly high [34]. Additionally, since they are already on a lot of medication and receiving treatment for their disability, receiving additional treatment for IVDD might be financially and physically burdensome. Providing targeted subsidies for disabled patients may help reduce financial barriers and encourage appropriate KMHC use. Patients with poorer perceived health status were more likely to use both KMHC and CMHC than CMHC only. This finding is consistent with previous research showing that individuals who perceive their health as poor tend to seek more active or diverse treatment options due to greater concern about their health [35]. Conversely, those already using both healthcare systems may perceive their health as poorer because of persistent or chronic symptoms. Therefore, potential reverse causality should be considered when interpreting these associations. Patients who engaged in regular physical activity were more inclined to use KMHC-only rather than CMHC-only for IVDD compared to patients who did not. Furthermore, patients who are concerned about their health and manage it via regular exercise might prefer KMHC. Patients with IVDD with shoulder joint or other spinal-related diseases such as spinal stenosis were more inclined to use KMHC-only relative to CMHC-only compared to those patients without such diseases. The KMHC—including acupuncture and herbal medicine—is actively employed globally in treating musculoskeletal disorders [36]. In Korea, musculoskeletal disorders remain a major disease condition that often causes visits to Korean medicine clinics [5,9]. Additionally, satisfaction with KMHC use for patients with musculoskeletal disorders is reported to be high [9,13].

No statistically significant differences were observed in the treatment contents and the annual number of healthcare uses of KMHC between KMHC-only users and those using both KMHC and CMHC. Both KMHC and CMHC users utilized conventional treatments more, including high-cost imaging tests such as MRI and computed tomography, intravenous injections, and physical therapy—compared to CMHC-only users. Additionally, although statistically insignificant, both KMHC and CMHC users tended to have higher copayments per visit for KMHC and CMHC, respectively, compared to those of KMHC-only and CMHC-only users. The higher medical expenses for KMHC among both KMHC and CMHC users might be attributed to the increased use of herbal decoction and pharmacopuncture, which are not covered by health insurance. Additionally, both KMHC and CMHC users utilized more high-cost imaging equipment compared to CMHC-only users, suggesting that the severity of IVDD might have been higher in both KMHC and CMHC users, necessitating more aggressive treatment. However, caution is needed in interpretation since the KHPS did not investigate the severity of IVDD and no information was provided on the amount of contribution of the insurer.

A key limitation of this study is that, as this study analyzed cross-sectional data from a single year, causal relationships cannot be inferred, and the associations identified could be bidirectional. Second, the KHPS dataset lacks detailed clinical information such as MRI grades, pain scales (e.g., Visual Analogue Scale scores), and disease duration, which may confound the observed associations. While we analyzed comorbid spine-related diseases and the use of high-cost imaging tests (MRI, CT), these cannot be considered adequate proxies for IVDD severity. Future studies incorporating objective clinical indicators of IVDD are warranted to better clarify these associations. Third, to prevent missing data related to medical use, patients were required to fill out a health household account book regarding their medical use. Additionally, various data, such as receipts for medical expenses and year-end settlements, were collected. However, it was challenging to determine the total medical expenses due to the absence of responses regarding the insurance burden for a significant number of patients. Future studies should analyze medical cost components such as patients’ copayments and insurance burden in detail to provide data with policy implications, thereby enhancing the applicability of the findings.

Nonetheless, given the high preference and widespread use of KMHC for IVDD [5,9,13], this study holds significance as the first to analyze RWE using nationwide representative sample data from Korea. The application of RWE in the medical field has recently expanded rapidly, supporting drug development, policy decisions, and treatment outcome analysis [15]. In Korea, dorsalgia, a primary symptom of IVDD, is among the most commonly treated symptoms with KMHC. However, patients with musculoskeletal disorders have expressed concerns about the expensive cost of KMHC [5,9]. Therefore, health insurance coverage for chuna manual therapy was extended to musculoskeletal diseases in 2019 in Korea. Furthermore, a pilot project is currently underway to expand health insurance coverage for herbal decoctions for lumbar disc herniation, reflecting public demand [9]. As health insurance coverage for KMHC in IVDD expands, the findings of this study can help policymakers make decisions related to the fair distribution of health insurance finances in the future. Additionally, the utilization status and factors of KMHC in patients with IVDD identified in this study can be utilized as variables and subgroup analysis plans when planning future clinical studies on IVDD.

In future studies, integrating longitudinal KHPS data or clinical registries would enable trend analyses and causal inference regarding KMHC utilization for IVDD. Moreover, combining KHPS data with other data sources, such as hospital registries or clinical databases, could facilitate the inclusion of objective indicators (e.g., MRI findings or pain scores), thereby improving the accuracy of disease severity assessment. Future research could include cost-effectiveness comparisons between KMHC and CMHC interventions and incorporate expected treatment durations to better contextualize clinical effectiveness. Establishing standardized KMHC protocols, including acupuncture frequency, session duration, and herbal formula components, would further enhance the reproducibility and practical applicability of the findings. Furthermore, extending this research to international contexts such as China or the United States—where traditional and complementary medicine utilization patterns differ [37,38]—could offer valuable comparative insights into policy integration and patient behavior.

## 5. Conclusions

The study identified significant demographic and health-related factors influencing the use of KMHC for IVDD. Specifically, individuals who were employed, had poorer perceived health, or had additional musculoskeletal conditions (e.g., shoulder or other spine diseases) were more likely to choose KMHC. These results provide insight into patient behavior and may help guide more effective healthcare planning and resource allocation for KMHC. Future research should employ longitudinal designs to clarify causal relationships and further evaluate the benefits of KHMC.

## Figures and Tables

**Table 1:** General characteristics of healthcare user groups with outpatient visits for intervertebral disc disease.

Variables	Total n (%)	CMHC-Only n (%)	Both KMHC and CMHC n (%)	KMHC-Only n (%)	*p* Value
Number of participants	495	305 (65.72) †	106 (21.76) †	84 (12.53) †	
Predisposing factors					
Sex					0.523
Men	188 (42.19)	118 (44.17)	44 (40.77)	26 (34.31)	
Women	307 (57.81)	187 (55.83)	62 (59.23)	58 (65.69)	
Age					0.030 *
19–44	64 (19.08)	35 (16.32)	20 (25.97)	9 (21.58)	
45–59	121 (40.71)	83 (47.10)	23 (29.62)	15 (26.43)	
60–74	202 (28.26)	131 (26.34)	41 (33.66)	30 (28.95)	
75 or older	108 (11.95)	56 (10.24)	22 (10.74)	30 (23.03)	
Region					0.045 *
Seoul/Incheon/Gyeonggi/Gangwon	154 (55.62)	107 (59.87)	33 (52.56)	14 (38.69)	
Daejeon/Sejong/Chungcheong	92 (10.97)	53 (8.79)	26 (15.72)	13 (14.18)	
Busan/Daegu/Ulsan/Gyeongsang	124 (19.81)	79 (20.48)	24 (16.12)	21 (22.68)	
Gwangju/Jeolla/Jeju	125 (13.60)	66 (10.86)	23 (15.60)	36 (24.45)	
Education level					0.212
Elementary school or below	170 (19.23)	104 (18.64)	30 (15.27)	36 (29.21)	
Middle/High school	211 (49.77)	132 (49.63)	50 (57.83)	29 (36.48)	
College or above	114 (31.00)	69 (31.72)	26 (26.89)	19 (34.31)	
Marital status					0.076
Married/Living together	363 (72.48)	232 (74.93)	80 (73.96)	51 (57.08)	
Widowed/Divorced/Separated/Never married	132 (27.52)	73 (25.07)	26 (26.04)	33 (42.92)	
Enabling factors					
Number of household members					0.267
1	79 (13.47)	42 (12.23)	14 (12.28)	23 (22.04)	
2	236 (29.71)	152 (28.76)	49 (34.02)	35 (27.18)	
3	71 (23.29)	45 (21.05)	14 (26.92)	12 (28.72)	
4 or more	109 (33.54)	66 (37.96)	29 (26.77)	14 (22.07)	
Household income					0.841
1st quartile (lowest)	115 (15.77)	68 (15.53)	18 (13.11)	29 (21.60)	
2nd quartile	136 (19.57)	81 (18.13)	37 (24.72)	18 (18.20)	
3rd quartile	116 (26.05)	76 (26.63)	21 (24.88)	19 (25.01)	
4th quartile (highest)	128 (38.61)	80 (39.71)	30 (37.29)	18 (35.20)	
Indemnity private health insurance					0.770
No	252 (33.63)	154 (33.29)	48 (31.98)	50 (38.25)	
Yes	243 (66.37)	151 (66.71)	58 (68.02)	34 (61.75)	
Employment status					0.327
Unpaid family worker/unemployed	221 (36.51)	146 (37.17)	38 (31.44)	37 (41.85)	
Employed	209 (52.22)	115 (49.63)	53 (60.32)	41 (51.74)	
Self-employed	65 (11.27)	44 (13.20)	15 (8.24)	6 (6.41)	
Need Factors					
Disability					0.071
No	439 (92.74)	267 (91.32)	99 (97.05)	73 (92.71)	
Yes	56 (7.26)	38 (8.68)	7 (2.95)	11 (7.29)	
Perceived health status					0.155
Very good/Good	104 (22.21)	72 (24.42)	16 (11.66)	16 (28.94)	
Fair	220 (47.27)	129 (47.53)	53 (49.12)	38 (42.72)	
Poor/Very poor	171 (30.52)	104 (28.05)	37 (39.22)	30 (28.34)	
Perceived stress					0.722
Barely	110 (16.28)	65 (16.24)	19 (13.08)	26 (22.06)	
Low	237 (50.54)	145 (50.64)	57 (54.62)	35 (42.92)	
High/Very high	148 (33.18)	95 (33.12)	30 (32.30)	23 (35.02)	
Depressed mood					0.780
No	451 (90.34)	275 (90.80)	98 (90.69)	78 (87.28)	
Yes	44 (9.66)	30 (9.20)	8 (9.31)	6 (12.72)	
Regular physical activities					0.046 *
No	224 (47.99)	151 (53.16)	39 (39.00)	34 (36.51)	
Yes	271 (52.01)	154 (46.84)	67 (61.00)	50 (63.49)	
Alcohol use					0.609
None	244 (40.00)	147 (37.30)	52 (45.25)	45 (45.01)	
Monthly or less	113 (25.95)	68 (27.12)	22 (20.66)	23 (29.01)	
2 or more times a month	138 (34.05)	90 (35.58)	32 (34.09)	16 (25.98)	
Cigarette use					0.262
No	437 (84.81)	265 (83.56)	93 (83.34)	79 (93.94)	
Yes	58 (15.19)	40 (16.44)	13 (16.66)	5 (6.06)	
BMI					0.593
<23	188 (38.46)	112 (37.28)	42 (40.40)	34 (41.32)	
23.0–24.9	130 (24.34)	83 (27.17)	28 (20.05)	19 (16.94)	
≥25.0	177 (37.20)	110 (35.56)	36 (39.55)	31 (41.73)	
Arthritis					0.664
No	357 (82.46)	219 (82.51)	82 (84.47)	56 (78.71)	
Yes	138 (17.54)	86 (17.49)	24 (15.53)	28 (21.29)	
Shoulder joint diseases					0.003 **
No	459 (94.58)	288 (95.87)	100 (96.25)	71 (84.90)	
Yes	36 (5.42)	17 (4.13)	6 (3.75)	13 (15.10)	
Other spine diseases					0.005 **
No	379 (84.62)	241 (85.76)	87 (89.70)	51 (69.80)	
Yes	116 (15.38)	64 (14.24)	19 (10.30)	33 (30.20)	
Number of chronic diseases					0.285
0	230 (55.68)	135 (53.94)	56 (59.30)	39 (58.50)	
1	156 (27.07)	107 (30.81)	29 (20.22)	20 (19.33)	
2 or more	109 (17.25)	63 (15.25)	21 (20.47)	25 (22.17)	

Notes: ** *p* < 0.01, * *p* < 0.05. The values represent unweighted frequency (weighted column proportion) for categorical variables. † represents the weighted row proportion for the number of participants. *p* values were obtained from Rao-Scott Chi-square tests for complex survey design. Abbreviations: BMI, body mass index; CMHC, conventional medicine healthcare; KMHC, Korean medicine healthcare.

**Table 2:** Factors associated with healthcare use in outpatient visits for intervertebral disc disease.

Variables	Crude Analysis		Fully Adjusted Model	
	(Both KMHC and CMHC) vs. CMHC-Only	KMHC-Only vs. CMHC-Only	(Both KMHC and CMHC) vs. CMHC-Only	KMHC-Only vs. CMHC-Only
	cOR (95% CI)	cOR (95% CI)	aOR (95% CI)	aOR (95% CI)
Predisposing factors				
Sex				
Men	1 [Reference]	1 [Reference]	1 [Reference]	1 [Reference]
Women	1.15 (0.63, 2.11)	1.51 (0.75, 3.08)	1.48 (0.69, 3.19)	1.35 (0.55, 3.34)
Age				
19–44	1 [Reference]	1 [Reference]	1 [Reference]	1 [Reference]
45–59	0.40 * (0.16, 0.96)	0.42 (0.14, 1.27)	0.28 * (0.09, 0.89)	0.49 (0.14, 1.77)
60–74	0.80 (0.36, 1.79)	0.83 (0.29, 2.34)	0.68 (0.16, 2.87)	1.40 (0.38, 5.11)
75 or older	0.66 (0.26, 1.69)	1.70 (0.61, 4.75)	1.15 (0.22, 5.99)	4.80 (0.92, 25.09)
Region				
Seoul/Incheon/Gyeonggi/Gangwon	1 [Reference]	1 [Reference]	1 [Reference]	1 [Reference]
Daejeon/Sejong/Chungcheong	2.04 (0.93, 4.44)	2.50 (0.81, 7.69)	2.50 * (1.04, 6.02)	2.59 (0.80, 8.38)
Busan/Daegu/Ulsan/Gyeongsang	0.90 (0.44, 1.85)	1.71 (0.73, 4.01)	1.20 (0.52, 2.74)	1.58 (0.56, 4.40)
Gwangju/Jeolla/Jeju	1.64 (0.73, 3.65)	3.48 ** (1.57, 7.70)	2.95 * (1.23, 7.09)	4.61 ** (1.67, 12.75)
Education level				
Elementary school or below	1 [Reference]	1 [Reference]	1 [Reference]	1 [Reference]
Middle/High school	1.42 (0.72, 2.82)	0.47 (0.22, 1.02)	1.81 (0.80, 4.08)	0.97 (0.39, 2.44)
College or above	1.03 (0.48, 2.23)	0.69 (0.31, 1.54)	0.99 (0.27, 3.55)	1.58 (0.45, 5.47)
Marital status				
Married/Living together	1 [Reference]	1 [Reference]	1 [Reference]	1 [Reference]
Widowed/Divorced/Separated/Never married	1.05 (0.55, 2.02)	2.25 * (1.10, 4.59)	0.71 (0.29, 1.71)	1.85 (0.57, 5.94)
Enabling factors				
Number of household members				
1	1 [Reference]	1 [Reference]	1 [Reference]	1 [Reference]
2	1.18 (0.49, 2.86)	0.52 (0.22, 1.26)	0.96 (0.28, 3.25)	0.78 (0.21, 2.99)
3	1.27 (0.44, 3.69)	0.76 (0.27, 2.09)	1.07 (0.28, 4.19)	2.14 (0.51, 8.96)
4 or more	0.7 (0.27, 1.82)	0.32 * (0.13, 0.82)	0.56 (0.12, 2.55)	0.82 (0.20, 3.45)
Household income				
1st quartile (lowest)	1 [Reference]	1 [Reference]	1 [Reference]	1 [Reference]
2nd quartile	1.62 (0.66, 3.98)	0.72 (0.29, 1.83)	1.56 (0.61, 4.02)	0.98 (0.35, 2.75)
3rd quartile	1.11 (0.42, 2.92)	0.68 (0.28, 1.63)	0.98 (0.33, 2.90)	1.17 (0.40, 3.42)
4th quartile (highest)	1.11 (0.45, 2.74)	0.64 (0.26, 1.53)	1.11 (0.39, 3.18)	1.31 (0.32, 5.40)
Indemnity private health insurance				
No	1 [Reference]	1 [Reference]	1 [Reference]	1 [Reference]
Yes	1.06 (0.58, 1.93)	0.81 (0.43, 1.52)	1.34 (0.52, 3.48)	1.73 (0.67, 4.46)
Employment status				
Unpaid family worker/unemployed	1 [Reference]	1 [Reference]	1 [Reference]	1 [Reference]
Employed	1.44 (0.75, 2.77)	0.93 (0.45, 1.90)	2.37 * (1.06, 5.30)	1.45 (0.53, 3.94)
Self-employed	0.74 (0.31, 1.76)	0.43 (0.14, 1.31)	0.99 (0.34, 2.89)	0.93 (0.21, 4.01)
Need Factors				
Disability				
No	1 [Reference]	1 [Reference]	1 [Reference]	1 [Reference]
Yes	0.32 * (0.11, 0.90)	0.83 (0.34, 2.01)	0.27 * (0.09, 0.81)	0.60 (0.20, 1.81)
Perceived health status				
Very good/Good	1 [Reference]	1 [Reference]	1 [Reference]	1 [Reference]
Fair	2.16 (0.89, 5.24)	0.76 (0.32, 1.79)	3.05 * (1.17, 8.00)	0.84 (0.29, 2.48)
Poor/Very poor	2.93 * (1.16, 7.41)	0.85 (0.34, 2.13)	6.13 ** (2.04, 18.45)	0.61 (0.20, 1.79)
Perceived stress				
Barely	1 [Reference]	1 [Reference]	1 [Reference]	1 [Reference]
Low	1.34 (0.62, 2.90)	0.62 (0.27, 1.42)	1.27 (0.57, 2.86)	1 (0.36, 2.73)
High/Very high	1.21 (0.52, 2.81)	0.78 (0.33, 1.85)	1.07 (0.42, 2.69)	1.51 (0.45, 5.04)
Depressed mood				
No	1 [Reference]	1 [Reference]	1 [Reference]	1 [Reference]
Yes	1.01 (0.40, 2.60)	1.44 (0.52, 4.01)	0.68 (0.26, 1.81)	1.25 (0.36, 4.31)
Regular physical activities				
No	1 [Reference]	1 [Reference]	1 [Reference]	1 [Reference]
Yes	1.77 (0.96, 3.29)	1.97 * (1.01, 3.84)	1.88 (0.98, 3.61)	2.65 * (1.19, 5.90)
Alcohol use				
None	1 [Reference]	1 [Reference]	1 [Reference]	1 [Reference]
Monthly or less	0.63 (0.28, 1.39)	0.89 (0.39, 2.02)	0.72 (0.31, 1.66)	1.05 (0.41, 2.67)
2 or more times a month	0.79 (0.40, 1.55)	0.61 (0.26, 1.39)	0.83 (0.38, 1.81)	0.68 (0.24, 1.92)
Cigarette use				
No	1 [Reference]	1 [Reference]	1 [Reference]	1 [Reference]
Yes	1.02 (0.43, 2.38)	0.33 (0.09, 1.15)	1.40 (0.55, 3.57)	0.49 (0.10, 2.47)
BMI				
<23	1 [Reference]	1 [Reference]	1 [Reference]	1 [Reference]
23.0–24.9	0.68 (0.32, 1.47)	0.56 (0.24, 1.32)	0.66 (0.29, 1.50)	0.65 (0.25, 1.70)
≥25.0	1.03 (0.52, 2.04)	1.06 (0.49, 2.3)	1.08 (0.53, 2.20)	1.62 (0.66, 3.97)
Arthritis				
No	1 [Reference]	1 [Reference]	1 [Reference]	1 [Reference]
Yes	0.87 (0.44, 1.69)	1.28 (0.65, 2.52)	0.76 (0.35, 1.63)	0.58 (0.22, 1.54)
Shoulder joint diseases				
No	1 [Reference]	1 [Reference]	1 [Reference]	1 [Reference]
Yes	0.91 (0.29, 2.85)	4.13 ** (1.56, 10.94)	1.26 (0.34, 4.68)	3.71 * (1.22, 11.29)
Other spine diseases				
No	1 [Reference]	1 [Reference]	1 [Reference]	1 [Reference]
Yes	0.69 (0.34, 1.42)	2.61 * (1.25, 5.44)	0.51 (0.21, 1.25)	2.63 * (1.16, 5.96)
Number of chronic diseases				
0	1 [Reference]	1 [Reference]	1 [Reference]	1 [Reference]
1	0.60 (0.29, 1.22)	0.58 (0.25, 1.34)	0.56 (0.24, 1.26)	0.51 (0.20, 1.29)
2 or more	1.22 (0.55, 2.71)	1.34 (0.63, 2.85)	1.17 (0.46, 2.95)	1.49 (0.59, 3.75)

Notes: ** *p* < 0.01, * *p* < 0.05. The values represent odds ratios with 95% confidence intervals for factors associated with healthcare use for intervertebral disc diseases. Crude analyses were performed using multinomial logistic regression to predict a multiclass dependent variable (users of KMHC-only, users of both KMHC and CMHC, and users of CMHC-only) using each individual independent variable among predisposing, enabling, and need factors. A fully adjusted model was obtained using a multinomial logistic regression model to predict a multiclass dependent variable using a combination of multiple independent variables which were predisposing, enabling, and need factors. Abbreviations: aOR, adjusted odds ratio; BMI, body mass index; CI, confidence interval; CMHC, conventional medicine healthcare; cOR, crude odds ratio; KMHC, Korean medicine healthcare.

**Table 3:** Korean medicine healthcare services for KHMC-only users and both KMHC and CMHC users.

Variables	Total	KMHC-Only	Both KMHC and CMHC	*p* Value
Number of participants	190	84	106	
Healthcare services				
Acupuncture	182 (92.81)	81 (91.93)	101 (93.32)	0.823
Moxibustion	14 (8.63)	5 (9.99)	9 (7.84)	0.711
Cupping therapy	59 (27.46)	31 (30.00)	28 (26.00)	0.641
Herbal decoction	33 (13.94)	13 (8.25)	20 (17.22)	0.073
Expensive herbal medicine preparations (such as Gongjindan)	1 (0.23)	1 (0.64)	0 (0)	NA
General herbal medicine preparations (such as granules and pills)	25 (10.33)	14 (12.99)	11 (8.81)	0.457
Pharmacopuncture	46 (27.35)	15 (19.95)	31 (31.61)	0.203
Chuna manual therapy	18 (13.74)	8 (12.49)	10 (14.46)	0.763
Manual therapy	11 (6.98)	2 (6.56)	9 (7.22)	NA
Physical therapy	172 (90.54)	71 (84.79)	101 (93.85)	0.202
Medical expenses of KMHC				
Copayment per visit (KRW/visit)	21,636 ± 2982	18,896 ± 4013	23,213 ± 4142	0.456
Day of healthcare uses				
Annual number of healthcare uses (times/year)	14.52 ± 1.91	14.38 ± 2.78	14.6 ± 2.54	0.953

Notes: The values represent unweighted frequency (weighted column proportion) for categorical variables and mean ± standard error for continuous variables. *p* values for complex survey design were obtained from Rao-Scott Chi-square tests for categorical variables, and general linear models analyses using sample weights for continuous variables. Abbreviations: CMHC, conventional medicine healthcare; KMHC, Korean medicine healthcare; KRW, Korean Won; NA, not applicable.

**Table 4:** Conventional medicine healthcare services for CMHC-only users and both KMHC and CMHC users.

Variables	Total	Both KMHC and CMHC	CMHC-Only	*p* Value
Number of participants	411	106	305	
Healthcare services				
High-cost imaging tests (MRI, CT, PET-CT)	99 (26.48)	36 (40.27)	63 (21.92)	0.010 *
Intravenous injections (such as fluids and nutritional injections)	46 (9.98)	19 (17.23)	27 (7.58)	0.027 *
Chemotherapy	1 (0.06)	0 (0)	1 (0.08)	NA
Manual therapy	44 (14.42)	9 (8.50)	35 (16.38)	0.090
Physical therapy	266 (66.41)	80 (78.51)	186 (62.41)	0.021 *
Vaccination	11 (1.64)	3 (1.96)	8 (1.53)	NA
Blood test or urine test	22 (5.54)	5 (4.52)	17 (5.87)	0.651
Medical expenses of CMHC				
Copayment per visit (KRW/visit)	51,953 ± 4355	55,589 ± 8376	50,749 ± 5099	0.621
Day of healthcare uses				
Annual number of healthcare uses (times/year)	10.32 ± 1.21	12.31 ± 3.11	9.66 ± 1.22	0.429

Notes: * *p* < 0.05. Details of the description for Table 4 were the same as those for Table 3. Abbreviations: CMHC, conventional medicine healthcare; CT, computed tomography; KMHC, Korean medicine healthcare; KRW, Korean Won; MRI, magnetic resonance imaging; PET, positron emission tomography.

## Data Availability

The data presented in this study are openly available in Korea Health Panel Survey (KHPS) at https://www.khp.re.kr:444/web/data/board/dataDownload.do?pageIndex=1&bbsid=107&keyField=&key=, accessed on 10 June 2024.

## Supplementary Materials

The following supporting information can be downloaded at: https://www.mdpi.com/article/10.3390/healthcare13212661/s1, Table S1: Factors associated with healthcare use in outpatient visits for intervertebral disc disease using predisposing and enabling factors.

## Abbreviations

The following abbreviations are used in this manuscript:

CMHC	Conventional medicine healthcare
DDD	Degenerative disc disease
IVDD	Intervertebral disc disease
KHPS	Korea Health Panel Survey
KMHC	Korean medicine healthcare
MRI	Magnetic resonance imaging
RWE	Real-world evidence
