# Supplementary material for: Real-World Evidence on the Use of Traditional Korean Medicine in Managing Intervertebral Disc Disease
Source: Healthcare (Basel). 2025 Oct 22;13(21):2661. doi: 10.3390/healthcare13212661 (PMC12607482; doi:10.3390/healthcare13212661)
Supplement: Supplementary file 1 [file healthcare-13-02661-s001.zip › healthcare-3895514-supplementary.pdf]

Table S1. Factors associated with healthcare use in outpatient visits for intervertebral disc disease using predisposing and enabling factors

Variables	Adjusted model 1		Adjusted model 2	
	(both KMHC and CMHC) vs. CMHC-only	KMHC-only vs. CMHC-only	(both KMHC and CMHC) vs. CMHC-only	KMHC-only vs. CMHC-only
	aOR (95% CI)	aOR (95% CI)	aOR (95% CI)	aOR (95% CI)
**Predisposing factors**				
Sex				
Men	1 [Reference]	1 [Reference]	1 [Reference]	1 [Reference]
Women	1.49 (0.79, 2.81)	1.76 (0.84, 3.71)	1.5 (0.74, 3.06)	1.53 (0.73, 3.21)
Age				
19–44	1 [Reference]	1 [Reference]	1 [Reference]	1 [Reference]
45–59	0.33* (0.12, 0.93)	0.52 (0.17, 1.66)	0.33* (0.12, 0.91)	0.56 (0.17, 1.79)
60–74	0.74 (0.26, 2.15)	1.14 (0.34, 3.83)	0.72 (0.21, 2.54)	1.66 (0.51, 5.38)
75 or older	0.84 (0.26, 2.72)	2.14 (0.64, 7.14)	1.06 (0.23, 4.86)	4.74* (1.11, 20.19)
Region				
Seoul/Incheon/Gyeonggi/Gangwon	1 [Reference]	1 [Reference]	1 [Reference]	1 [Reference]
Daejeon/Sejong/Chungcheong	1.73 (0.79, 3.81)	2.22 (0.71, 6.89)	1.83 (0.83, 4.01)	2.66 (0.84, 8.39)
Busan/Daegu/Ulsan/Gyeongsang	0.87 (0.41, 1.86)	1.61 (0.65, 3.99)	0.97 (0.45, 2.05)	1.83 (0.71, 4.72)
Gwangju/Jeolla/Jeju	1.87 (0.78, 4.49)	3.72** (1.57, 8.77)	2.18 (0.91, 5.25)	5.37*** (2.13, 13.5)
Education level				
Elementary school or below	1 [Reference]	1 [Reference]	1 [Reference]	1 [Reference]
Middle/High school	2.21* (1.03, 4.73)	1.3 (0.51, 3.31)	2.02 (0.9, 4.55)	1.02 (0.41, 2.53)
College or above	1.42 (0.49, 4.14)	2.02 (0.73, 5.55)	1.24 (0.37, 4.14)	1.45 (0.47, 4.44)
Marital status				
Married/Living together	1 [Reference]	1 [Reference]	1 [Reference]	1 [Reference]
Widowed/Divorced/Separated/Never married	0.87 (0.42, 1.82)	1.7 (0.83, 3.49)	0.87 (0.36, 2.11)	1.76 (0.61, 5.06)
**Enabling factors**				
Number of household members				
1			1 [Reference]	1 [Reference]
2			1.14 (0.32, 4.04)	0.8 (0.25, 2.57)
3			1.37 (0.31, 6.08)	2 (0.53, 7.61)
4 or more			0.7 (0.14, 3.52)	0.76 (0.22, 2.68)
Household income				
1st quartile (lowest)			1 [Reference]	1 [Reference]
2nd quartile			1.49 (0.53, 4.18)	1.04 (0.41, 2.65)
3rd quartile			0.93 (0.3, 2.91)	1.07 (0.39, 2.91)
4th quartile (highest)			1.1 (0.36, 3.3)	1.45 (0.44, 4.76)
Indemnity private health insurance				
No				
Yes			1 [Reference]	1 [Reference]
Employment status				

Unpaid family worker/unemployed	1 [Reference]	1 [Reference]
Employed	1.89 (0.82, 4.38)	1.27 (0.52, 3.09)
Self-employed	0.94 (0.34, 2.6)	0.6 (0.17, 2.15)

Notes: ****P* < .001, ***P* < .01, **P* < .05.

The values represent odds ratios with 95% confidence intervals for factors associated with healthcare use for intervertebral disc diseases. Adjusted model 1 employed a multinomial logistic regression model to predict a multiclass dependent variable using predisposing factors. Adjusted model 2 used a similar model, incorporating both predisposing and enabling factors.

Abbreviations: aOR, adjusted odds ratio; BMI, body mass index; CI, confidence interval; CMHC, conventional medicine healthcare; cOR, crude odds ratio; KMHC, Korean medicine healthcare.
